# Dosimetric effects of the acuros XB and anisotropic analytical algorithm on volumetric modulated arc therapy planning for prostate cancer using an endorectal balloon

**DOI:** 10.1186/s13014-015-0346-3

**Published:** 2015-02-22

**Authors:** Taeryool Koo, Jin-Beom Chung, Keun-Yong Eom, Jin-Yong Seok, In-Ah Kim, Jae-Sung Kim

**Affiliations:** Department of Radiation Oncology, Seoul National University College of Medicine, 103 Daehak-ro, Jongno-gu, Seoul, 110-799 Korea; Department of Radiation Oncology, Seoul National University Bundang Hospital, 82 Gumi-ro, 173beon-gil, Bundang-gu, Seongnam-si, 463-707 Korea

**Keywords:** Acuros XB, Anisotropic analytical algorithm, Endorectal balloon, Prostate cancer, Volumetric modulated arc therapy

## Abstract

**Background:**

To compare the dosimetric effects of Acuros XB (AXB) and Anisotropic Analytical Algorithm (AAA) on volumetric modulated arc therapy (VMAT) planning for postoperative prostate cancer patients irradiated using an endorectal balloon (ERB).

**Methods:**

We measured central axis doses with film in a phantom containing an air cavity, and compared measurements with calculations of the AAA and AXB. For clinical study, 10 patients who had undergone whole pelvic radiotherapy (WPRT) followed by prostatic bed-only radiotherapy (PBRT) using VMAT were enrolled. An ERB was used for PBRT but not for WPRT. To compare dosimetric parameters, the cumulative dose-volume histograms, mean, maximum, and minimum doses were measured for the planning target volume. Homogeneity of plans were confirmed using V_95%_, V_107%_ (V_X%_, percentage volumes receiving at least X% of prescribed doses) and conformity indices (homogeneity index [HI], conformity index [CI], and conformation number [CN]). We compared volumes of the organ-at-risk receiving 10% to 100% (10-tier at 10% interval) of prescribed doses (V_10%_ – V_100%_).

**Results:**

In the phantom study, the AAA showed larger disagreement with the measurements, and overestimated the dose in the air cavity, comparing with the AXB. For WPRT planning, the AAA predicted a lower maximum dose and V_107%_ than the AXB. For PBRT planning, the AAA estimated a higher minimum dose, lower maximum dose, and smaller V_107%_, and larger V_95%_ than the AXB. Regarding the conformity indices, the AAA was estimated to be more homogenous than the AXB for PBRT planning (HI, 0.088 vs. 0.120, p = 0.005; CI, 1.052 vs. 1.038, p = 0.022; and CN, 0.920 vs. 0.900, p = 0.007) but not for WPRT planning. Among V_10%_ to V_100%_ of the rectum, the PBRT exhibited significant discrepancies in V_30%_, V_40%_, V_70%_, V_80%_, and V_90%_; while the WPRT did in V_20%_ and V_30%_.

**Conclusions:**

The phantom study demonstrated that the AXB calculates more accurately in the air cavity than the AAA. In the clinical setting, the AXB exhibited different dosimetric distributions in the VMAT plans for PBRT containing an ERB. The AXB should be considered for prostate cancer patients irradiated with an ERB for better applying of heterogeneous condition.

**Electronic supplementary material:**

The online version of this article (doi:10.1186/s13014-015-0346-3) contains supplementary material, which is available to authorized users.

## Background

After radical prostatectomy, postoperative (adjuvant or salvage) radiotherapy is recommended for patients with adverse pathological features or biochemical failure. However, the extent of radiotherapy—in other words, irradiation of the whole pelvis or prostate bed only—remains controversial. Although the results of randomized trials about the extent of radiotherapy have not been reported, several retrospective studies have shown a benefit of whole pelvic radiotherapy (WPRT) in terms of biochemical progression-free survival [1,2]. WPRT encompasses more pelvic organs than does prostatic bed-only radiotherapy (PBRT); hence, adverse effects on the genitourinary and gastrointestinal systems are a primary concern. However, intensity-modulated radiotherapy (IMRT) can deliver a higher dose to the target and lower doses to critical organs [3,4]. Recently, volumetric modulated arc therapy (VMAT), a next-generation IMRT technique, was introduced. VMAT has been reported to feature a shorter delivery time and a plan quality at least comparable to that of fixed-field IMRT in WPRT [5] and PBRT [6,7].

At our institution, postoperative radiotherapy is performed as follows: WPRT without an ERB followed by PBRT with an ERB via VMAT. The endorectal balloon (ERB) was used with its efficiency for prostate cancer treatment. The use of ERB has been found to be well-tolerated and effective in reducing the intrafraction motion and improving the sparing of rectal wall by reducing the rectal volume in the high-dose region, resulting in significant reduction in rectal toxicity [8-12]. However, the ERB is an air cavity and thus renders the pelvic cavity a heterogeneous area. Tissue heterogeneity should be corrected to ensure accurate dose calculations, especially in small volumes such as the prostate [13,14].

The Anisotropic Analytical Algorithm (AAA; Varian Medical Systems, Palo Alto, CA, USA), a commonly used convolution-superposition algorithm, reflects heterogeneity and thus has been proven to provide better dose calculation results [9,15]. However, the AAA is also known to overestimate the dose at the air-tumour interface because it incorporates only the density differences in the heterogeneous media in dose computations [16]. When an ERB is used to reduce the intrafraction motion, the prostatic bed is located adjacent to the air cavity; therefore, the AAA might yield an inaccurate dose calculation. Recently, the Acuros XB Advanced Dose Calculation (AXB) was released in conjunction with the Eclipse treatment planning system (Varian Medical Systems). The AXB solves the linear Boltzmann Transport Equation, and has known similarities to the Monte Carlo method (MC). In several studies with heterogeneous media, the AXB has been reported to estimate dose deposition more accurately than AAA [17-19].

To our knowledge, only 1 previous study has compared the dose distributions of AXB and AAA in prostate cancer and found no difference between the results [20]. However, the above-mentioned study did not used ERB, so a heterogeneous area created by ERB was not thoroughly accounted. Therefore, it is needed to compare these differences in more heterogeneous media, which can result from an ERB. The aim of this study was to compare the dosimetric impact of AXB and AAA on VMAT planning for postoperative prostate cancers treated with an ERB.

## Methods

### Verification in a phantom with an air cavity

**Figure 1:**
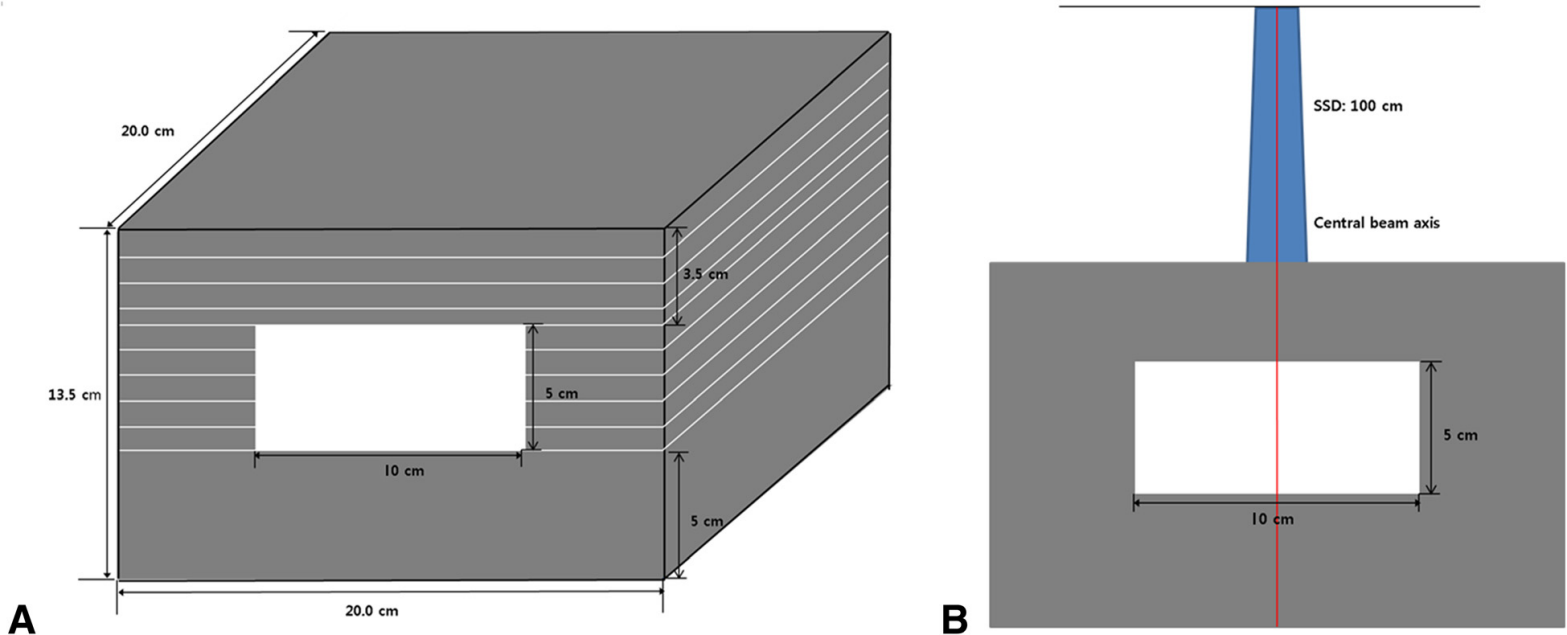
**Geometric diagram of (A) the phantom with an air cavity and (B) experimental setup for calculation and measurement of the central axis dose.**

As shown in the Figure 1, a rectangular acryl phantom with an air cavity was manufactured specially for this study. The phantom’s overall dimensions were 20 × 20 × 13.5 cm^3^, and included an air cavity (10 × 20 × 5 cm^3^). The phantom was scanned with the computed tomography (CT; The Brilliance CT Big Bore, Philips, Netherlands) simulator. The reconstructed digital imaging and communication in medicine (DICOM) CT data were then transferred to the Eclipse. The central axis dose (CAD) was calculated from AXB and AAA for 5 × 5 cm^2^ fields of 6 and 10 MV beams. All dose calculations were performed to deliver 2 Gy to the isocenter at depth of maximum dose. To evaluate the accuracy of the AXB and AAA, the CADs were measured by using radiochromic films (GafChromic EBT3, International Specialty Products, NJ, USA) at 2 cm depth of homogeneous zone and various depths (4.5, 5.5, 6.5, 7.5 and 8.5 cm) of air cavity within the phantom. The measured CADs were then compared with the calculations from AXB and AAA. For film dosimetry, a calibration curve of film was obtained at the dose levels from 0 to 5 Gy. The check of linear accelerator output was performed with ion chamber by applying the TG 51 protocol [21].

### Patient selection and simulation

Ten prostate cancer patients who had undergone radical prostatectomy between October 2013 and May 2014 were enrolled in the current planning study, which was approved by our institutional review board. All patients were treated with WPRT followed by PBRT. A CT simulation was performed while the patients were placed in a knee-feet fix (CIVCO, Orange City, IA, USA) in a supine position on a flat couch. The patients were asked to drink 300 ml of water 1 hour prior to the simulations for both WPRT and PBRT to ensure that the bladder was completely filled. For the PBRT simulation, an ERB manufactured in our institution (Additional file 1: Figure S1) was inserted into the rectum and filled with 70 cc of air. After 1 minute, the ERB catheter was placed at the pre-marked position, and the inflated ERB was immobilized above the anal sphincter. Detailed process of the ERB system was reported in our previous study [12].

### Target delineation

For WPRT, the prostatic bed and regional lymph node area, including the presacral, obturator, and external and internal iliac lymph nodes, were contoured as the clinical target volume (CTV). The planning target volume (PTV) included the CTV plus an additional margin of 0.5 cm in all directions, except the superior and inferior directions, where a 1-cm margin was added. For PBRT, only the prostatic bed was delineated as the CTV, and the PTV was defined as the CTV plus a 1-cm margin in all directions except posterior to the prostatic bed, where a 0.7-cm margin was added. The rectum and bladder were contoured as the organs at risk (OARs). The rectum was contoured to extend from the sigmoid flexure to the bottom of the ischium.

### Treatment planning and dosimetric parameter evaluation

Eclipse version 11.0.34 (Varian Medical Systems) was used to generate the VMAT plans using 10-MV photon beams from a Varian TrueBeam STx equipped with a high-definition 120-multileaf collimator in the dynamic mode at 600 monitor units (MU)/minute. For WPRT, we used 2 rotating full arcs: the first arc rotated clockwise from 181 to 179 degrees with a 30-degree collimator rotation and the second arc rotated counter-clockwise from 179 to 181 degrees with a 330-degree collimator rotation. For PBRT, 2 full arcs were used in the same directions; the degrees of collimator rotation were 15 and 345 for the clockwise and counter-clockwise directions, respectively. The isocenter was the center of the PTVs.

The prescription doses were 44 Gy for WPRT followed by 22 Gy for PBRT, administered in daily doses of 2 Gy. The goal of optimization was to ensure that ≥95% of the PTV should receive 100% of the prescribed dose and that 107% of the prescribed dose should be restricted to ≤5% of the PTV. The dose constraints of the OARs are listed in Table 1. All VMAT plans were calculated using both the AAA and AXB algorithms. Two dose-reporting modes are available in the AXB: dose-to-water (*D*_*w*_) and dose-to-medium (*D*_*m*_); the latter mode was selected. For the AXB, version 11.0 of the physics material table was used. For all plans, the optimization was an automatic intermediate dose calculation. The dose calculation grid size was set at 2.5 mm for all cases.

**Table 1:** **Dose volume constraints adopted for planning study**

**Radiotherapy**	**Organs at risk**	**Constraints**
Whole pelvis (44 Gy/22 fractions)	Rectum	V_33 Gy_ < 50%
		V_39.6 Gy_ < 30%
		V_44 Gy_ < 20%
	Bladder	V_30.8 Gy_ < 70%
		V_39.6 Gy_ < 50%
		V_44 Gy_ < 30%
Prostatic bed (22 Gy/11 fractions)	Rectum	V_11 Gy_ < 40%
		V_15.4 Gy_ < 25%
		V_19.8 Gy_ < 10%
	Bladder	V_11 Gy_ < 35%
		V_15.4 Gy_ < 25%
		V_19.8 Gy_ < 20%

Cumulative dose-volume histograms (DVH) and parameters were calculated for all cases. The average DVH of PTV was generated for the AAA and AXB plans by averaging the data over the 10 analyzed cases. The mean doses, maximum doses, and minimum doses to the PTV were measured. To represent the target coverage and hot areas, the PTV receiving more than 95% (V_95%_) and 107% (V_107%_) of the prescribed dose were evaluated. Several conformity indices were analysed to evaluate plan homogeneity.

First, the homogeneity index (HI) of the PTV (as defined by the International Commission on Radiation Units and Measurements, report 83 [22]) was calculated using Equation (1).

1 $$ \mathrm{H}\mathrm{I}=\frac{\left({\mathrm{D}}_{2\%}-{\mathrm{D}}_{98\%}\right)}{{\mathrm{D}}_{50\%}} $$

D_2%_ represents the maximum dose received by 2% of the PTV, D_98%_ represents the minimum dose received by 98% of the PTV, and D_50%_ represents the dose received by 50% of the PTV. A lower HI indicates a more homogeneous plan because D_2%_ and D_98%_ are surrogate markers of the maximum and minimum doses to the PTV, respectively.

Second, the conformity index (CI) as defined by the Radiation Therapy Oncology Group was calculated using Equation (2).

2 $$ \mathrm{C}\mathrm{I}=\frac{{\mathrm{V}}_{\mathrm{RI}}}{\mathrm{TV}} $$

V_RI_ is the volume of reference isodose, and TV is the PTV volume. A CI equal to 1 corresponds to an ideal conformation, whereas a CI >1 indicates the irradiation of healthy tissues [23].

Lastly, the conformation number (CN) was evaluated to consider the irradiation of healthy tissue. The CN is the product of 2 fractions.

3 $$ \mathrm{C}\mathrm{N}=\frac{{\mathrm{TV}}_{\mathrm{RI}}}{\mathrm{TV}}\times \frac{{\mathrm{TV}}_{\mathrm{RI}}}{{\mathrm{V}}_{\mathrm{RI},}} $$

TV and TV_RI_ represent the PTV volume and the volume covered by the reference isodose line, respectively. The first fraction indicates the quality of target coverage and the second fraction represents the volume of healthy tissue irradiated with the reference isodose or higher [24]. In the present study, we used the 95% isodose as the reference isodose.

The mean doses, maximum doses, and minimum doses to the OARs were recorded. We compared volumes of the OAR receiving categorized doses, which the prescribed dose was divided into 10 ranges at 10% intervals, from V_10%_ to V_100%_. Additionally, DVHs of OARs were plotted.

Furthermore, differences of total MUs between the 2 dose calculation algorithms were compared. The Eclipse treatment planning system was used in this study, including both the AXB and AAA algorithms. It was installed on a standard clinical workstation (Dell® Precision T5500) with dual 2.4-GHz quad-core Intel processors E5620, 24-GB RAM memory, and a 64-bit Windows 7 operating system.

The Wilcoxon rank test was used to evaluate the statistical significance of differences between the AAA and AXB. Differences were considered to be statistically significant at a *p*-value <0.05. All statistical tests were performed using Predictive Analytics Software, version 18.0 (SAP America, Inc., Newtown Square, PA, USA).

## Results

### Verification of the central axis dose in the phantom with an air cavity

**Figure 2:**
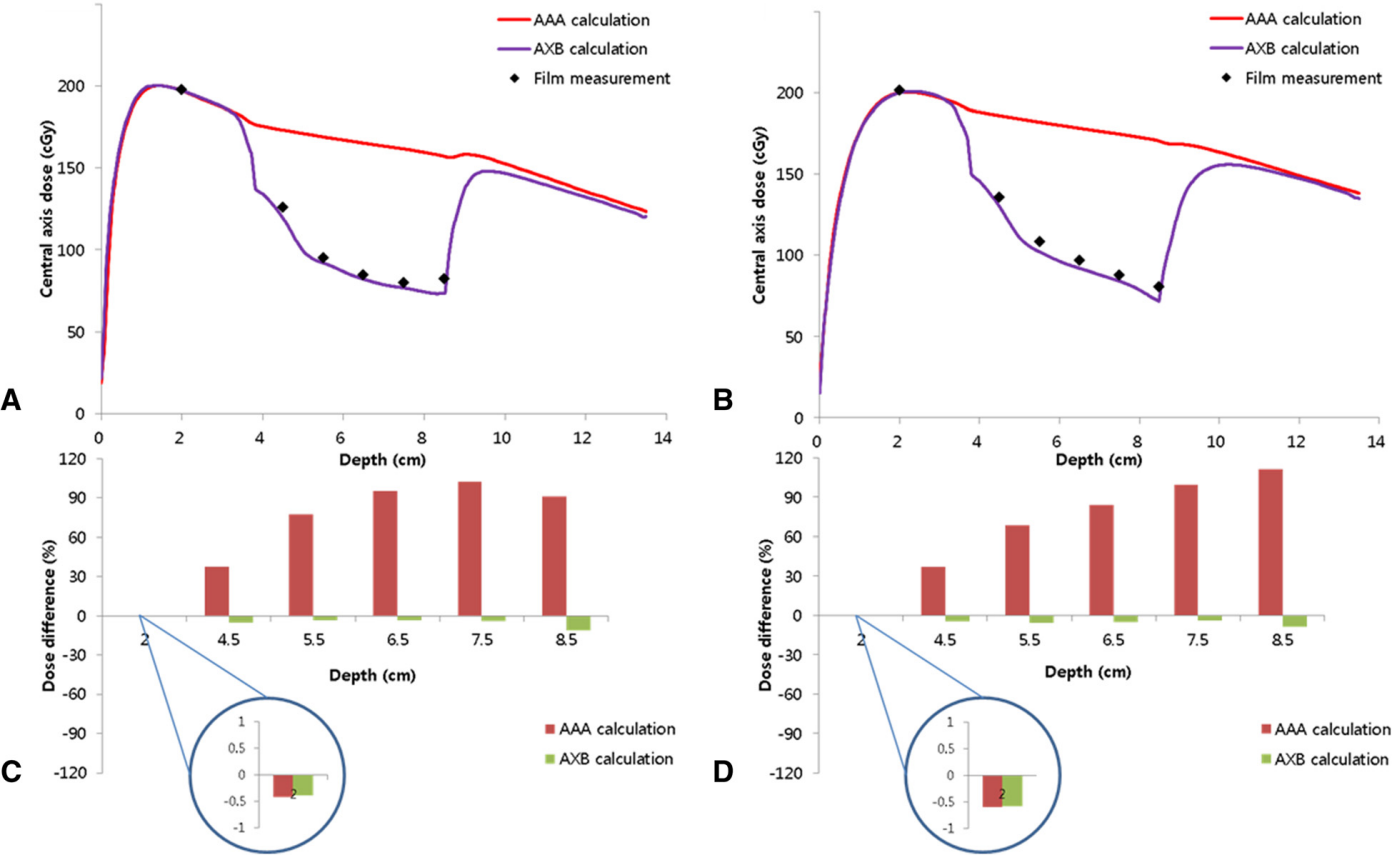
**The central axis depth dose curves calculated by AAA and AXB compared to the measured data using film for 5 × 5 cm** ^**2**^ **fields of (A) 6 MV and (B) 10 MV beams; the percentage differences between calculations and measurements for (C) 6 MV and (D) 10 MV beams at depths of 2, 4.5, 5.5, 6.5, 7.5, and 8.5 cm.**

The CAD curves for 5 × 5 cm^2^ field incident on the phantom with air cavity are shown in Figure 2, for 6 and 10 MV. The percentage differences of calculated CAD for both algorithms, relative to the film measurements, are displayed in the same figure at depths of 2, 4.5, 5.5, 6.5, 7.5, and 8.5 cm.

For 6 and 10 MV, The percentage differences of CAD calculated by both algorithms in homogeneous region (enlarged circles in Figure 2) were within 1.0% relative to CAD by film measurement. Excellent agreement was found between measurement and both algorithms. Although taking little underestimation in air cavity, the CAD by AXB was found to be in agreement with film measurement, whereas the AAA results showed higher CAD to this region compared to the measurements. The AAA overestimated the dose up to 102.7% for 6 MV and 99.6% for 10 MV. Similar trend were observed in region of an air-tissue interface. Overall, for AXB, the difference between the measured and calculated CAD ranged from −0.3% to 10.9% for 6 MV and from −0.6% to 8.9% for 10 MV. The AAA had wider range of difference between calculation and measurements, from −0.4% to 102.7% for 6 MV and from −0.6% to 111.3% for 10 MV.

### DVH analysis for the PTV

**Figure 3:**
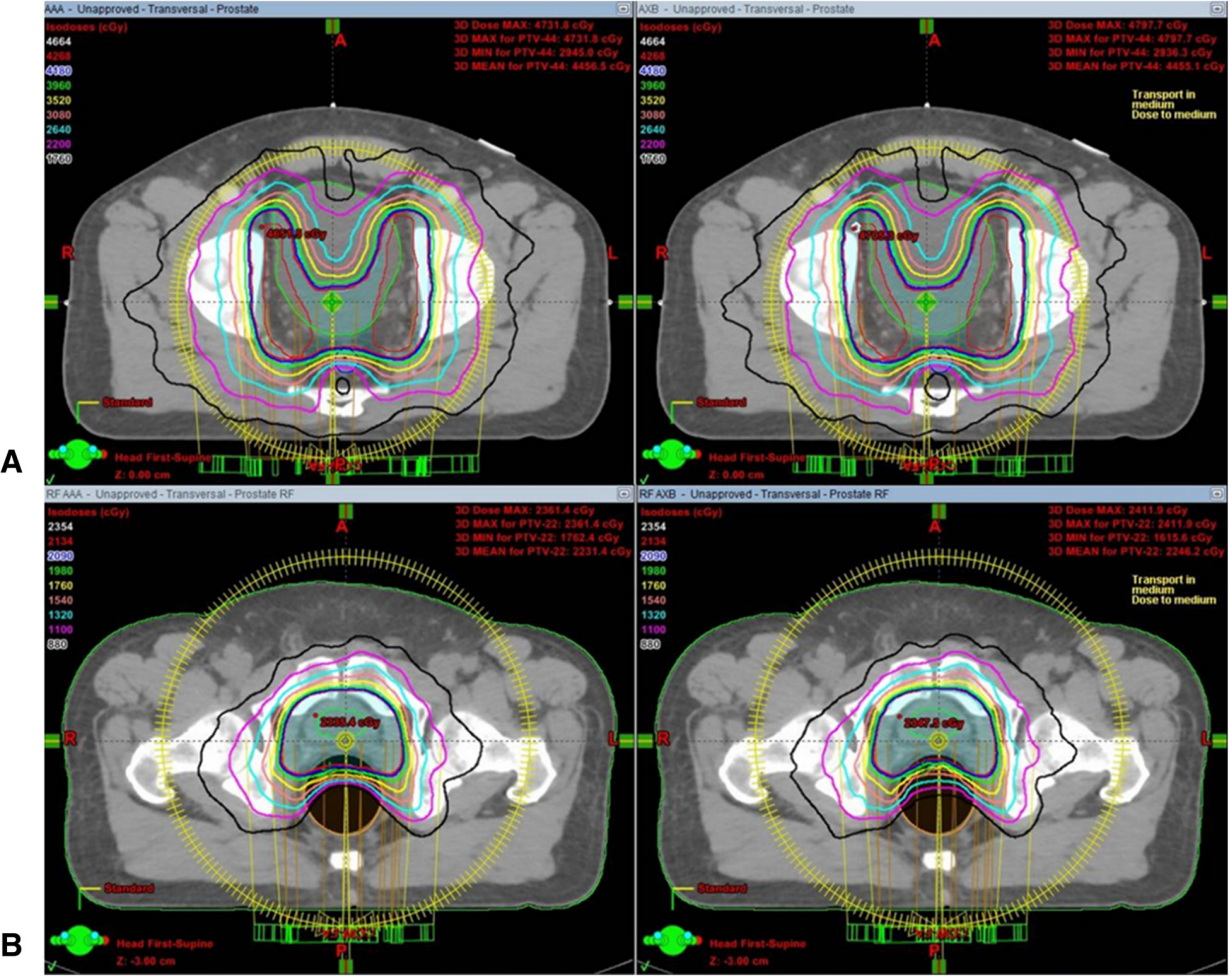
**Isodose curves for the applied anisotropic analytical algorithm and Acuros XB plans in patients from (A) whole pelvic radiotherapy and (B) prostatic bed-only radiotherapy.**

**Figure 4:**
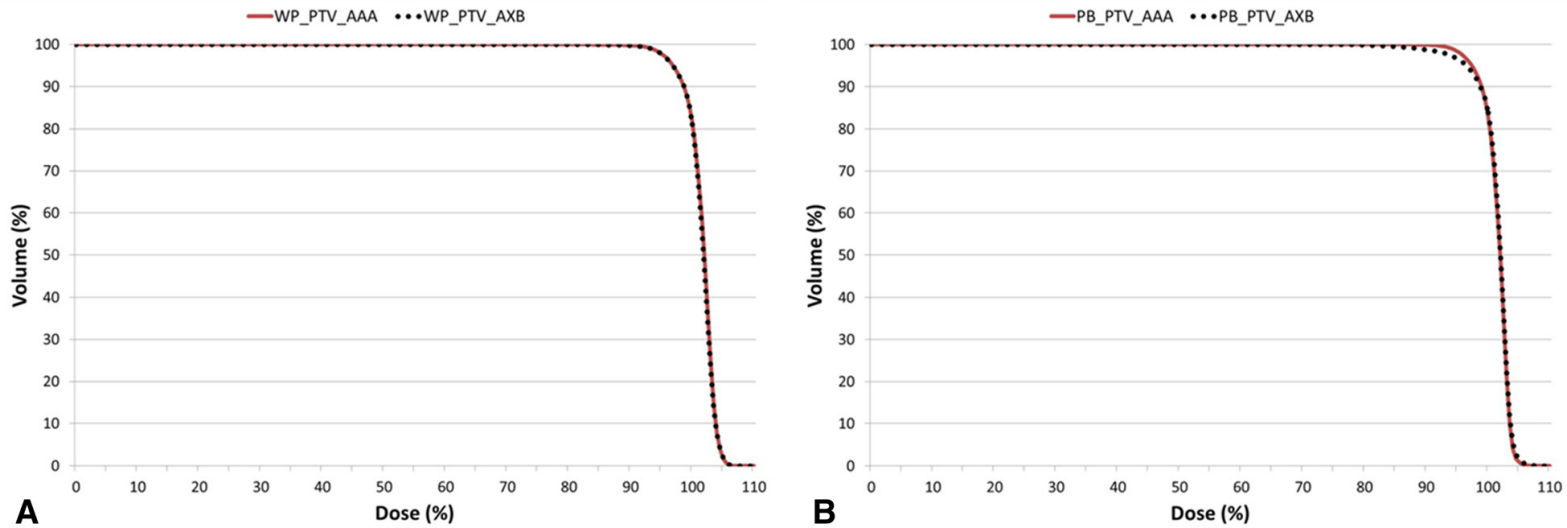
**The cumulative dose-volume histograms of the planning target volumes (PTVs) in the AAA (solid line) and AXB (dotted line) plans averaged over the 10 analysed patient’s plans for (A) the whole pelvic radiotherapy and (B) the prostatic bed only radiotherapy.**

The mean volumes of the PTVs were 780.87 ± 90.55 cm^3^ and 160.64 ± 34.05 cm^3^ for WPRT and PBRT, respectively. The isodose curves for the applied AAA and AXB plans are presented in Figure 3. The detailed dosimetric parameters are listed in Table 2. For the WPRT plans, a small but significant difference was observed in the maximum doses to the PTV; specifically, the AAA dose was lower than the AXB dose (4742.09 cGy vs. 4792.10 cGy, p = 0.005). The minimum dose was higher with the AAA, but this difference was not statistically significant (3330.66 cGy vs. 3259.04 cGy, p = 0.285). V_107%_, representing the volume with higher dose, was significantly smaller with the AAA than with the AXB (0.01% vs. 0.03%, p = 0.005). In contrast, no difference in the V_95%_ was observed between AAA and AXB (97.77% and 97.78%, p = 0.878). The average DVH of the PTV for the AAA and AXB plans is plotted in Figure 4A, which shows similar curves for WPRT.

**Table 2:** **Target coverage, homogeneity, and conformity indices of the planning target volume**

	**Whole pelvic radiotherapy**			**Prostatic bed-only radiotherapy**		
	**AAA**	**AXB**	**p**	**AAA**	**AXB**	**p**
Maximum Dose (cGy)	4742.09 ± 22.80	4792.10 ± 19.04	0.005	2377.51 ± 17.18	2399.08 ± 14.92	0.028
Minimum Dose (cGy)	3330.66 ± 226.58	3259.04 ± 226.00	0.285	1887.38 ± 133.54	1687.91 ± 218.88	0.005
Mean Dose (cGy)	4465.49 ± 13.67	4463.18 ± 11.16	0.037	2233.12 ± 8.17	2230.54 ± 7.94	0.333
95% Isodose Volume (cm^3^)	819.53 ± 128.25	820.56 ± 128.71	0.037	168.96 ± 35.32	166.83 ± 36.14	0.022
V_107%_ (%)	0.01 ± 0.01	0.03 ± 0.02	0.005	0.04 ± 0.04	0.13 ± 0.10	0.005
V_100%_ (%)	81.81 ± 3.00	81.08 ± 2.82	0.013	83.19 ± 4.34	83.85 ± 2.44	0.959
V_95%_ (%)	97.77 ± 0.43	97.76 ± 0.49	0.878	98.40 ± 0.76	96.62 ± 1.10	0.005
D_2%_ (%)	104.75 ± 0.44	104.88 ± 0.36	0.017	104.42 ± 0.38	104.69 ± 0.33	0.161
D_98%_ (%)	94.86 ± 0.50	94.86 ± 0.59	0.859	95.49 ± 0.83	92.46 ± 1.92	0.005
Homogeneity Index	0.097 ± 0.007	0.098 ± 0.008	0.059	0.088 ± 0.007	0.120 ± 0.019	0.005
Conformity Index	1.047 ± 0.071	1.048 ± 0.071	0.059	1.053 ± 0.041	1.038 ± 0.031	0.022
Conformation Number	0.916 ± 0.049	0.915 ± 0.049	0.203	0.920 ± 0.026	0.900 ± 0.028	0.007
First Factor	0.978 ± 0.004	0.978 ± 0.005	0.878	0.984 ± 0.008	0.966 ± 0.011	0.005
Second Factor	0.937 ± 0.052	0.936 ± 0.052	0.074	0.935 ± 0.030	0.932 ± 0.026	0.241

AAA = Anisotropic Analytical Algorithm; AXB = Acuros XB.

For the PBRT plans, the AAA estimated an 11% higher minimum dose (1887.38 cGy vs. 1687.91 cGy, p = 0.005) as well as a 1% lower maximum dose (2377.51 cGy vs. 2399.08 cGy, p = 0.028). Additionally, the V_107%_ and V_95%_ differed significantly between the plans; specifically, the AAA yielded a smaller V_107%_ (0.04% vs. 0.13%, p = 0.005) and larger V_95%_ (98.40% vs. 96.62%, p = 0.005) relative to the AXB. The AAA and AXB curves showed obvious difference around V_95%_ for PBRT planning (Figure 4B).

### Conformity indices for the PTV

For WPRT, no difference was found in the conformity indices between AAA and AXB. However, for PBRT, all of the conformity indices were significantly different. The AAA yielded a lower HI (0.088 vs. 0.120, p = 0.005) and higher CI (1.053 vs. 1.038, p = 0.022) relative to the AXB. Similarly, the CN was higher with the AAA. When the first and second factors were calculated separately, the first factor was found to be the main contributor to the difference in the CN (0.984 and 0.966 for AAA and AXB, respectively, p = 0.005), whereas the second factor was similar for the AAA and AXB (0.935 and 0.932, respectively, p = 0.241).

### DVH analysis for the OARs

**Figure 5:**
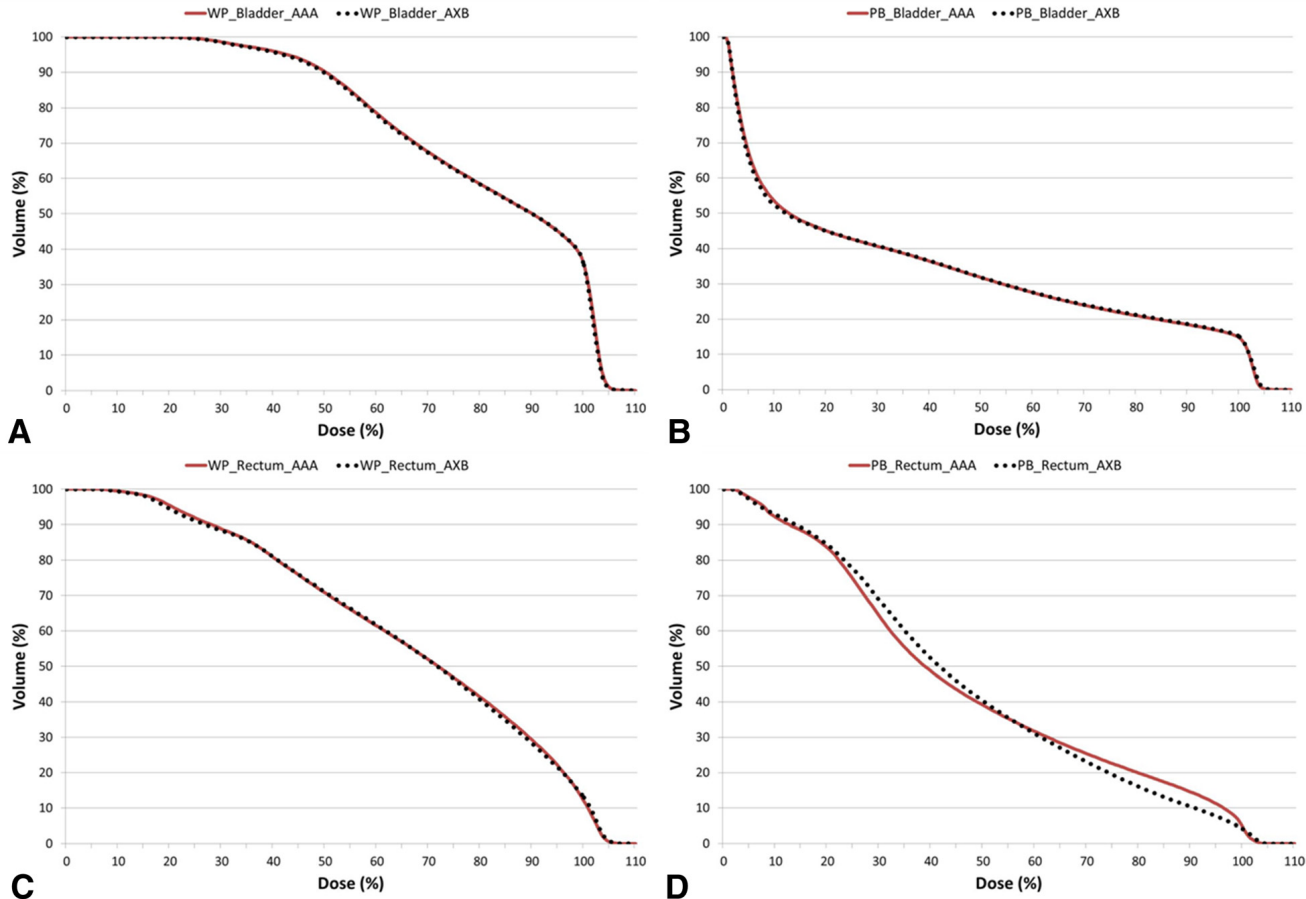
**The cumulative dose-volume histograms of the organs at risk in the AAA (solid line) and AXB (dotted line) plans averaged over the 10 analysed patient’s plans: (A) bladder in the whole pelvic radiotherapy, (B) bladder in the prostatic bed only radiotherapy, (C) rectum in the whole pelvic radiotherapy, and (D) rectum in the prostatic bed only radiotherapy.**

The mean doses, maximum doses, and minimum doses to the OARs are listed in Table 3. The maximum and minimum doses to the rectum exhibited the most significant differences between the AAA and AXB, regardless of the radiation field size. The average DVHs were quantified as V_10%_ to V_100%_ of the OARs (Table 3) and plotted in Figure 5. For the rectum, statistically significant discrepancies were found more in PBRT (V_30%_, V_40%_, V_70%_, V_80%_, and V_90%_) than WPRT (V_20%_ and V_30%_; Figure 5D).

**Table 3:** **Dosimetric data of organs at risk**

**Bladder**	**WP**	**Volume**	**314 ± 117.52 cm** ^**3**^											
		**Maximum dose (cGy)**	**Minimum dose (cGy)**	**Mean dose (cGy)**	**V10% (%)**	**V20% (%)**	**V30% (%)**	**V40% (%)**	**V50% (%)**	**V60% (%)**	**V70% (%)**	**V80% (%)**	**V90% (%)**	**V100% (%)**
AAA	Mean	4747.15	1266.97	3595.53	100.00	99.97	98.59	96.02	90.12	78.44	67.44	58.39	49.97	35.52
	(SD)	149.54	403.32	284.69	0.00	0.08	2.62	6.12	7.83	9.52	11.31	12.95	14.26	13.70
AXB	Mean	4732.93	1236.55	3585.11	100.00	99.96	98.49	95.71	89.69	77.93	67.11	58.24	49.95	35.48
	(SD)	172.23	388.58	287.13	0.00	0.10	2.76	6.13	7.89	9.52	11.31	13.00	14.32	13.71
	p	0.114	0.059	0.005	1	0.109	0.138	0.012	0.007	0.005	0.005	0.047	0.959	0.646
**Bladder**	**PB**	**Volume**	**278.8 ± 109.23 cm** ^**3**^											
		**Maximum dose (cGy)**	**Minimum dose (cGy)**	**Mean dose (cGy)**	**V10% (%)**	**V20% (%)**	**V30% (%)**	**V40% (%)**	**V50% (%)**	**V60% (%)**	**V70% (%)**	**V80% (%)**	**V90% (%)**	**V100% (%)**
AAA	Mean	2365.24	35.61	776.31	53.23	45.04	40.66	36.44	31.78	27.51	23.91	20.98	18.42	14.61
	(SD)	21.69	34.74	280.98	17.47	15.81	15.21	14.96	13.98	12.58	11.14	9.94	8.92	7.56
AXB	Mean	2377.19	32.77	775.61	52.16	45.01	40.74	36.52	31.84	27.58	24.05	21.19	18.60	14.96
	(SD)	32.42	32.39	284.16	17.25	15.85	15.30	15.03	14.04	12.66	11.27	10.10	9.08	7.90
	p	0.114	0.005	0.646	0.007	0.093	0.799	0.646	0.508	0.444	0.047	0.007	0.013	0.285
**Rectum**	**WP**	**Volume**	**44.47 ± 19.44 cm** ^**3**^											
		**Maximum dose (cGy)**	**Minimum dose (cGy)**	**Mean dose (cGy)**	**V10% (%)**	**V20% (%)**	**V30% (%)**	**V40% (%)**	**V50% (%)**	**V60% (%)**	**V70% (%)**	**V80% (%)**	**V90% (%)**	**V100% (%)**
AAA	Mean	4650.79	546.54	2991.27	99.45	95.35	88.80	80.75	70.69	61.36	51.85	41.34	29.25	11.98
	(SD)	55.88	178.89	337.26	1.18	4.25	8.93	10.28	11.61	13.17	13.29	11.31	9.51	7.18
AXB	Mean	4694.80	516.34	2981.62	99.39	94.36	88.32	80.86	70.96	61.61	51.75	40.53	28.22	12.96
	(SD)	58.69	169.66	347.89	1.30	5.22	9.37	10.51	11.76	13.31	13.57	11.91	10.01	7.14
	p	0.028	0.013	0.386	0.109	0.007	0.013	0.203	0.575	0.386	0.878	0.508	0.241	0.093
**Rectum**	**PB**	**Volume**	**108.26 ± 13.7 cm** ^**3**^											
		**Maximum dose (cGy)**	**Minimum dose (cGy)**	**Mean dose (cGy)**	**V10% (%)**	**V20% (%)**	**V30% (%)**	**V40% (%)**	**V50% (%)**	**V60% (%)**	**V70% (%)**	**V80% (%)**	**V90% (%)**	**V100% (%)**
AAA	Mean	2300.09	80.53	1041.95	92.07	83.50	64.17	48.57	39.04	31.64	25.34	19.90	14.54	4.66
	(SD)	22.59	35.67	115.34	7.34	10.20	10.26	6.94	5.65	5.77	5.93	5.54	4.59	2.50
AXB	Mean	2331.40	76.05	1038.99	92.83	84.35	68.70	52.15	40.24	30.99	23.02	16.03	10.36	4.15
	(SD)	30.64	34.04	108.82	5.29	8.07	8.55	7.66	6.66	6.56	6.02	4.68	2.92	1.42
	p	0.007	0.005	0.646	0.169	0.799	0.009	0.007	0.093	0.285	0.005	0.005	0.005	0.333

WP = Whole pelvis; AAA = Anisotropic Analytical Algorithm; AXB = Acuros XB; SD = Standard deviation.

### Technical parameters

The percentage differences in the MUs for the prostate VMAT plans calculated with the 2 algorithms are listed in Table 4. The average percentage differences in the total MU between the 2 dose calculation algorithms were 0.19% for WPRT and 3.54% for PBRT.

**Table 4:** **The average monitor units between two dose calculation algorithms on volumetric modulated arc therapy plans for all patients**

**Treatment**		**AAA (Avg ± SD)**	**AXB (Avg ± SD)**	**Relative difference (%)**
Whole pelvic radiotherapy	Monitor Unit	523 ± 45	524 ± 45	0.19
Prostatic bed-only radiotherapy	Monitor Unit	508 ± 29	526 ± 33	3.54

AAA = Anisotropic Analytical Algorithm; AXB = Acuros XB; Avg = Average, SD = Standard deviation.

## Discussion

The AAA accounts for tissue inhomogeneity by independently assuming the primary beam direction and lateral direction, whereas divergence from the upper level is not considered. Tissue properties are not included in the AAA, either. In contrast, the AXB considers the movement and interactions of radiation within the heterogeneous media and calculates the doses to each voxel according to the tissue properties. Hence, the AXB can more accurately predict dose distribution, compared with the AAA [25,26].

Rana et al. [20] used Rapid Arc plans to perform a planning study of prostate cancer patients in which the clinical dosimetric impacts of AAA and AXB were compared. In that study, the AAA predicted higher minimum and maximum doses to the PTV (range, 0.05–0.43%) than did the AXB, although this difference was not statistically significant. Similarly, the V_100_ (the percentage of PTV covered by 100% of the prescribed dose) values obtained with AAA (95%) and AXB (range, 93.1–97.9%) did not significantly differ. Although statistical significance was not evaluated, the AAA predicted higher doses to the majority of points (average, 94%) in a point-dose difference analysis. The authors concluded that there were no significant differences between AAA and AXB.

In contrast, according to our DVH analysis for PBRT, the AAA yielded significantly different dose predictions; in particular, the minimum dose was higher and the maximum dose lower than those obtained with the AXB. Similarly, for WPRT, the AAA yielded a lower maximum dose and tended to predict a higher minimum dose. The V_107%_ values, or so-called hot spots, were larger when calculated using the AXB for both the WPRT and PBRT plans. Another dose-volume parameter, the V_95%_ value, was larger with AAA for the PBRT plans; however, the values were similar for the WPRT plans. These differences in the dose-volume parameters might indicate differences in the target coverage predictions of AAA and AXB.

To compare PTV coverage in greater detail, we analysed several conformity indices. For the WPRT plans, the AAA and AXB did not significantly differ with respect to the CI, CN, and HI values. However, for the PBRT plans, we observed significant differences in the conformity indices. First, the HI values were lower with the AAA than with the AXB. A lower HI value indicates more homogeneous target coverage, and therefore, the AAA could be expected to underestimate heterogeneity in the PBRT plans. Second, the AAA was found to have a higher CI value, indicating that the V_95%_ in the PBRT plans was overestimated by the AAA. Third, a discrepancy was found in the CN values, particularly in the first part of the equation, which defines the quality of target coverage. Therefore, conformity indices analysed in the current study implied that heterogeneity of the PBRT fields was less reflected by the AAA than the AXB.

In the PBRT plans, the dosimetric discrepancies between the AAA and AXB were likely influenced by the ERB. Because the ERB generates an air cavity in the rectum, the resulting heterogeneity should be corrected in order to accurately predict the dose distribution in the prostatic bed. According to our DVH analysis for the rectum, the AAA and AXB exhibited significant differences in 5 ranges in the PBRT plan and in 2 ranges in the WPRT plan. These discrepancies were confirmed in the average DVH plots for the rectum (Figure 5D). The average DVH plots for other OARs did not differ significantly between the WPRT and PBRT plans.

Previous studies that compared the clinical impact of AXB and AAA in patients with non-small cell lung cancer [19,27] or nasopharyngeal cancer [26] reported that the dose distributions differed beyond the air cavity. In heterogeneous phantom studies, the dosimetric result of the AXB was more similar to MC than to the AAA [17,18,28]. In a heterogeneous interface-containing phantom, some differences between both algorithms were pointed out at interface between different materials and the doses calculated by AXB were significantly lower at the air-tissue interface than were those calculated by the AAA. Relative to the MC calculation, the AXB exhibited ±2% agreement, whereas the AAA exhibited a greater difference of up to 17.5% [18]. In a measurement study with inhomogeneous phantoms containing air gap, measured data were compared with doses calculated by the AAA and AXB [29]. According to this study, the AXB showed less discrepancies (−3.81% to + 0.9%) with measured data than the AAA (−3.1% to − 10.9%). However, in an air cavity, the difference between measured and calculated data was not mentioned in this study.

In our phantom study, film measurements were performed in an air cavity and the air-tissue interface. Our verification results showed that the calculated results of AXB had better agreement in air cavity and air-tissue interface than that of AAA when compared to the measured data. The results of film measurement, of course, showed actually little larger than that of AXB. The reason may be mainly due to film’s property which has the tissue-equivalent density. We found that dosimetric results in and around heterogeneous medium may be affected by the inclusion of the dosimeter, leading to an error in dose estimate. Therefore, care should be taken when performing measurements with film in phantom containing an air cavity.

The above-mentioned planning study conducted by Rana et al. [20], which compared the AAA and AXB in the context of prostate cancer patients, differed from our study in several aspects. First, a partial single-arc technique was used to avoid beam entrance through the couch-rails. Second, an ERB was not used. Consequently, it was possible that the heterogeneity due to the air cavity in the rectum had little influence on dose distribution. In contrast, we concurrently used 2 full arcs and an ERB; hence, the AAA and AXB exhibited differences in the predicted dose distributions. Additionally, Rana et al. [20] used same number of MUs for AAA and AXB, different from our study. In particular, the differences in MUs were more prominent in the PBRT plans, up to 3.54%. Similarly, Khan et al. [30] reported that the AXB required more MUs (average 2%) compared with AAA. This can be explained that more MUs were required by the increase of heterogeneity region in PTV due to use of an ERB.

In the current study, calculated rectal dose had statistically significant difference between the AAA and AXB, but the difference was somewhat small in absolute value. This may be because we prescribed relatively low dose for the PBRT planning. With the elevated dose, the absolute difference would be more marked.

## Conclusions

Our phantom study demonstrated that the AXB is significantly more accurate for dose calculation in the region of air cavity and air-tissue interface than the AAA, when compared to the measured data. For comparison of 10 patients with prostate cancer, the AXB and AAA yielded significantly different dosimetric distributions for VMAT plans with 2 full arcs for prostate cancer. In particular, several conformity indices were significantly different for the PBRT plans. These differences likely resulted from the ERB being used for PBRT, which was located posterior to the prostatic bed and rendered the radiation field a heterogeneous area. The differences in the dose-volume parameters for the rectum were remarkable in the PBRT plans generated with AXB and AAA. The AXB should be considered rather than the AAA for prostate cancer patients irradiated with an ERB for better applying of heterogeneous condition and precise analysing of rectal dose.

## Additional file

Additional file 1: Figure S1. An example of endorectal balloon made in our institution: (A) the endorectal balloon and its applications and (B) inflated balloon with 70 cc of air.
