# Supplementary figures and images for: Dosimetric effects of the acuros XB and anisotropic analytical algorithm on volumetric modulated arc therapy planning for prostate cancer using an endorectal balloon
Source: Radiat Oncol. 2015 Feb 22;10:48. doi: 10.1186/s13014-015-0346-3 (PMC4342085; doi:10.1186/s13014-015-0346-3)

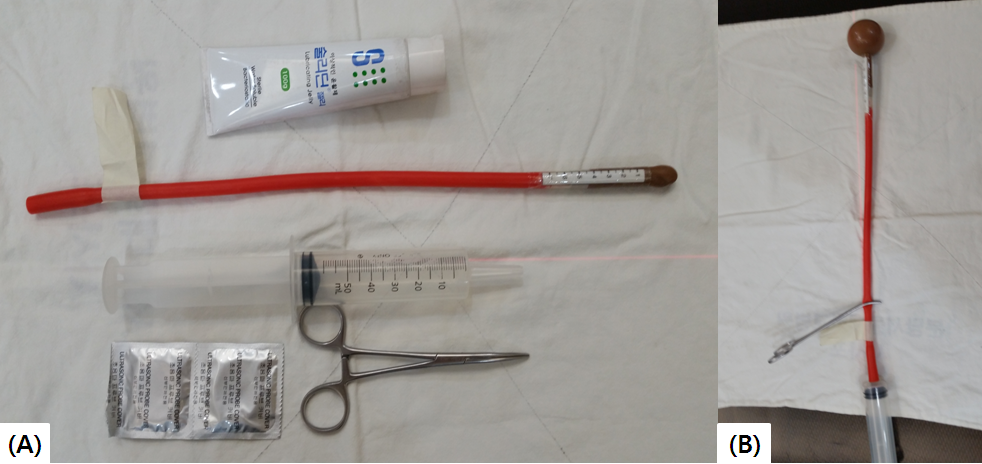

Supplement: Additional file 1: Figure S1. — An example of endorectal balloon made in our institution: (A) the endorectal balloon and its applications and (B) inflated balloon with 70 cc of air. [file 13014_2015_346_MOESM1_ESM.tif]
